# Cleaning and Smoothing Palatine Surfaces of Rubber Plates

**Published:** 1873-12

**Authors:** B. M. Wilkerson


### ARTICLE V.

### *Cleansing and Smoothing Palatine Surface of Rubber Plates.*

BY B. M. WILKERSON, M. D., D. D. S.

After removing a rubber plate from the flask, it is to be cleaned, which can be much facilitated by using a small wire brush. All the plaster and roughness can be readily removed from the palatine surface where it is difficult to reach with any other instrument. The brush should be made of very fine brass wires, bound together with a stout wire, (like the file cleaner,) in a bundle not exceeding an eighth of an inch in diameter. This is a convenient instrument in the laboratory for other purposes, and a trial will ensure its use.

---

### SELECTED ARTICLES.

---

